# A quantitative analysis of the structure of human sternum

**DOI:** 10.4103/0971-6203.51934

**Published:** 2009

**Authors:** Azim Arbabi

**Affiliations:** Department of Medical Physics, Imam Hosein Hospital, Shahid Beheshti Medical University, P. O. Box 14335-1419, Tehran, Iran

**Keywords:** Bone, bone structure, dosimetry, omnidirectional path length, sternum, trabecular bone

## Abstract

An extensive study of the human sternum has been carried out to obtain estimates of the omnidirectional path-length distributions and structural parameters for trabeculation and marrow spaces. Data for sternum samples have been collected, using an object plane scanning microscope. These data have been used to produce the omnidirectional path-length distributions and values of structural parameters for the whole sternum. For a typical adult man the mean trabecular and marrow space path lengths are 224 and 1364 *μ*m, respectively. The percentage bone volume is 13.8 and the surface to volume ratio is 190 cm. Data on the structural variations within the whole sternum are presented. They show a percentage difference in bone volume between the manubrium and the body of sternum of about 36%.

## Introduction

The sternum is frequently subjected to external radiations from diagnostic radiography (chest X rays) and radiotherapy in the chest area (e.g. in the treatment of breast cancer) and is also at risk from internal radiation by various bone-seeking radio nuclides.[1] For accurate radiation dosimetry with regard to radiation protection,[2] diagnostic radiology and radiotherapy, knowledge of the trabecular structure of the human sternum is required.

Hitherto these data have not been available, and hence an extensive study of the sternum as a whole has been performed to determine the omnidirectional path-length distribution and certain structural parameters of trabecular bone within the sternum.[3]

Analysis is complicated by the anisotropic nature of the trabeculation.[4,5] Consequently, the generation of representative omnidirectional path-length distributions necessitates the sampling of a randomized set of sectioning planes and scan directions.[6] Determination of the omnidirectional path-length distributions and structural parameters for the sternum are based on sets of scans in a limited number of directions and planes within each structure. Scans were obtained using an object-plane scanning microscope that has been described in detail elsewhere.[7,8] The only bone site that has been quantitatively analyzed and considered by many investigators is the lumbar vertebral body.[9] Lumbar vertebrae are less difficult to obtain than other bones and their convenient size and shape make it easy to prepare sections and consequently to calculate the omnidirectional path-length distributions and other structural parameters. Darley[10] studied a wide range of lumbar vertebra from individuals of both sexes aged from birth to 85 years. Beddoe *et al*.[7] extended the investigation and analyzed the biological variations of the lumbar vertebra structural with age up to 92 years. Beddoe[12] also studied the trabecular structure of beagle, miniature pig and rhesus monkey. Structural data have also been obtained for some other human bone sites such as long bones, rib, ilium and cranium, by the Leeds bone dosimetry group,[7,11] and Hildebrand *et al*., though often only one specimen from each bone site had been scanned.

## Materials and Methods

The techniques used for sample preparation are those described by Beddoe *et al*.,[7] with some improvements and modifications.[8] The bones analyzed in the present work were all fresh and unfixed samples. They were obtained from postmortem Caucasian subjects who had not been bed-ridden and who had died of causes unrelated to mineral metabolism.[6] No relevant reported data were available on the structure of the sternum that could be used for comparison. The third lumbar vertebra of the same cadaver was also obtained,[11] where possible, and analyzed to serve as a reference sample and to help confirm that the sternum could be categorized as normal. The results for the lumbar vertebra were compared with corresponding data quoted in the field of bone dosimetry by Darley[10] and Beddoe.[12,13]

**Figure 1:**
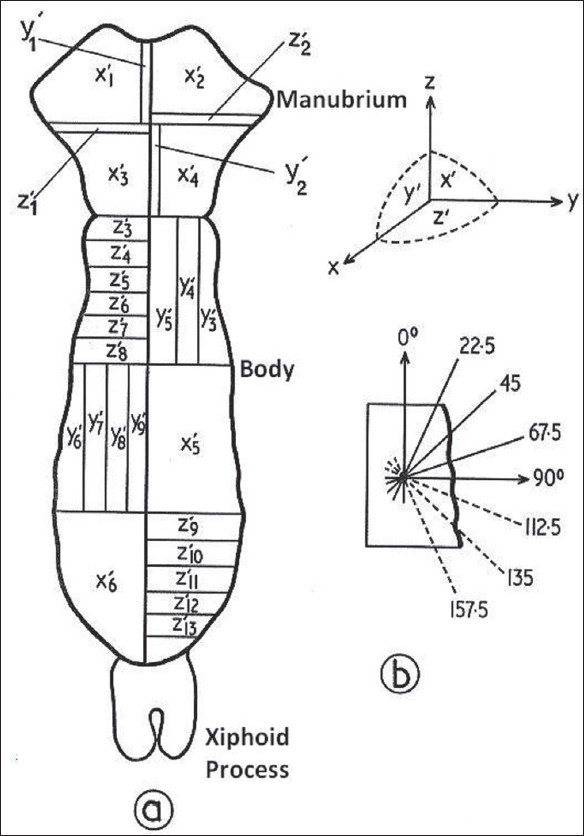
Slicing and scanning plans for the sternum (symmetry investigation)

Slices of thickness 3-6 mm were taken in the three orthogonal planes X1, Y1 and Z1 as shown in Figure 1, and the areas of the slices were kept as large as possible appropriate to the scan field size.

**Figure 2:**
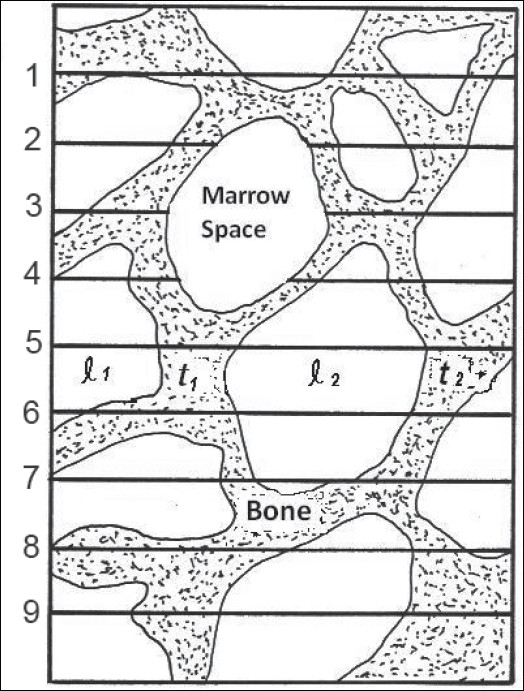
Scan pattern of trabecular (t) and marrow space (l) path lengths

A 30-*μ*m section such as Figure 2 was obtained from the center of each slice to represent the whole section from which it was drawn. Microradiographs were obtained from the 30-*μ*m sections. Any cortical bone was excluded by appropriate masking, and scanning was carried out using the object-plane scanning microscope. The xiphoid process, because of its small size and thickness (< 0.5 cm), and hence negligible amount of trabeculation, was excluded from all the path-length measurements.

### Omnidirectional Path-Length Distributions

**Figure 3:**
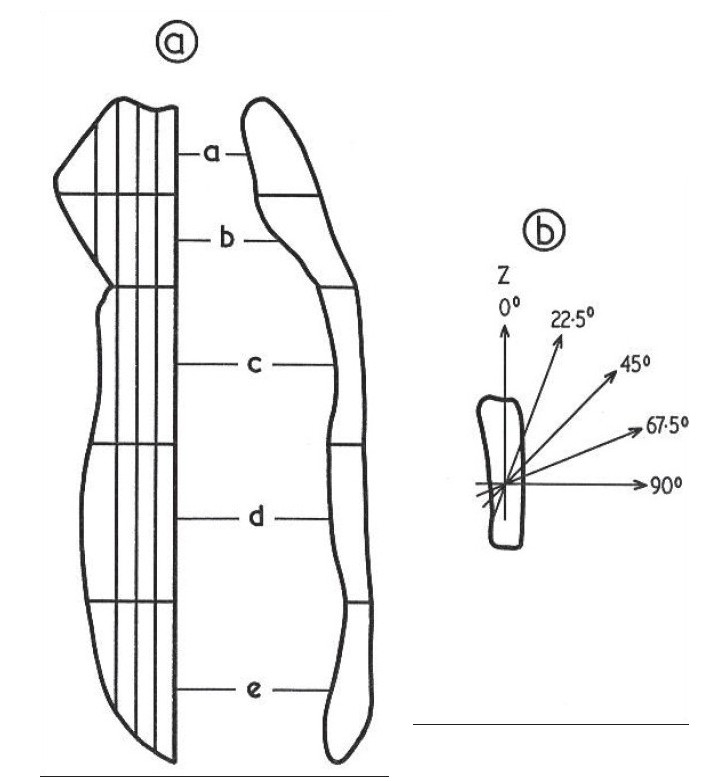
Slicing and scanning plans for the sternum to produce omnidirectional path length distributions

In a bone that possesses symmetry of alignment about the vertical (Z) axis, the scanning measurements in any set of sections cut parallel to the symmetry axis are sufficient to produce an omnidirectional distribution representative of the whole bone. Figure 1 shows the slicing design used to investigate the sternum for symmetry of alignment. It was verified that the symmetry of alignment about the vertical axis is consistent throughout the whole volume of sternum. Consequently, in analysis of the human sternum sets of parallel, sagittal sections of equal thickness were scanned and used to produce an omnidirectional path-length distribution for the whole bone. In practice, a whole sternum was cut sagittally from the jugular notch to the xiphi-sternal junction, to produce two exact halves. Structurally, one half simulates the other, and hence one-half suffices for structural analysis. The half used for structural analysis was cut sagittally into five sets of 3-6 mm thick slices as indicated in Figure 3a.

**Figure 4:**
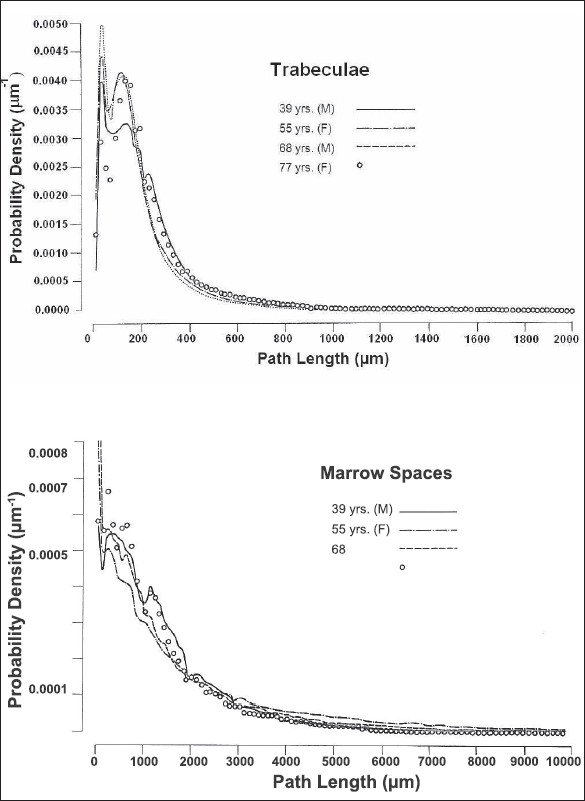
Omnidirectional path length distributions, human adults' sternum

To produce omnidirectional path-length distributions, 30-*μ*m-thick sections from each slice were scanned in five directions at 22.5° intervals, as shown in Figure 3b. These were found, by extensive scanning, to be adequate for obtaining a representative omnidirectional distribution of path lengths. The Leeds Automatic Bone Scanner (LABS) was used in the present investigation. For this work extensive reconstruction and recalibration have been made to the scanner, originally designed and constructed by Darley (1968 and 1972). For scanning a radiograph of a bone section is rotated on a turntable beneath a projection microscope and is scanned by a light beam. The radiograph substage moves radially across the turntable, at the same time rotation takes place, such that the light beam entering the microscope scans a series of arcs of constant radius across the bone. The magnified image of each scan is focused on an aperture, the reverse side of which is viewed by a photomultiplier. The duration of a light pulse entering the photomultiplier then measures the arc length traversed across a trabeculae (or marrow space), which does not differ significantly from the linear path length, because the scanning radius is very large compared with the length measured. The output pulses produced by the photomultiplier are proportional to the corresponding path lengths across the light features (marrow spaces or trabeculae, Figure 2) and are stored in a multichannel analyzer to produce a frequency distribution of path lengths. The average or general distribution can be calculated by normalizing and calculating the mean of the distributions of all the scans. To produce omnidirectional distributions, volume and solid-angle weightings were applied to the scans. The volume weighting factor is proportional to the relative marrow space or trabecular volume that is represented by the slice, and the solid-angle weighting allows for the fact that only particular directions in the structure are scanned. The omnidirectional path-length distributions for the four adult sternums studied are shown in Figure 4 for trabeculae and marrow spaces.

Structural parameters: Four structural parameters have been derived for the sternum: the mean marrow space path length (l), mean trabecular path length (t), percentage bone volume (Bv) and the endosteal surface to volume (of the mineralized component) ratio (S/V). The endosteal tissues lining the medullary cavity of a bone are radiobiologically important. The values of these structural parameters are frequently referred to by authors to specify the structural changes in bone with age or to reveal inter-species variations.[14–18] Mean trabecular and marrow space path lengths are used for calculation of mean marrow and endosteal doses for bones incorporating radioisotopes.[19,20] Percentage bone volume (Bv), [volume of the mineralized component/volume of the whole bone] × 100, is of special relevance in the study of bone structure in certain other contexts such as osteoporosis.

The percentage bone volume is also important in mineral metabolism studies.[21–24] The surface-to-volume ratio of bone is important in relation to mineral metabolism, but is of particular interest in the field of bone dosimetry for surface-seeking radioisotopes. Calculations of the relative toxicity of these isotopes compared with those that deposit throughout the whole volume of bone must include a factor for the surface to volume ratio.[16,20] Details of the sternums and vertebrae analyzed are presented in Table 1. The values obtained for the omnidirectional mean trabecular and marrow space path lengths are given with their respective standard errors, together with percentage bone volume and the surface to volume and marrow space mean path lengths are on average within ±7%.

**Table 1:** Structural parameters for sternums and lumbar vertebrae

*Age (years)*	*2*	*3*	*4*	*5*	*6*	*7*
	
	*Sex*	*Bone*	*t ± S.E. (μm)*	*ℓ ±S.E (μm)*	*BV*	*S/Vcm-1*
39	Male	Sternum	224 ± 17	1364 ± 71	13.8	190
		LV3	233 ± 17	1139 ±84	15.4	184
55	Female	Sternum	183 ± 13	1825 ± 128	8.8	242
		LV3	184 ± 18	1339 ± 116	10.9	251
68	Male	Sternum	176 ± 10	1505 ± 116	10.2	265
77	Female	Sternum*	227 ± 16	1333 ± 112	16.2	198

* A central sagittal slice, 1.5 cm wide from manubrium running lengthwise, with 2 cm below the manubrium in the body of sternum only.

In the case of the sternums of a 39-year-old man and 55-year-old woman, their corresponding third lumbar vertebral has also been analyzed for comparison purposes. Table 1 presents the results for these subjects showing that the mean trabecular path lengths in sternums and the third lumbar vertebrae are very close (within ±4%). In contrast, the mean marrow space path lengths are on average 28% larger in sternums than in their corresponding third lumbar vertebrae. These findings justify smaller values of percentage bone volume for sternums than for the third lumbar vertebrae (average 18%), and thus relatively more bone marrow in the sternum than in the third lumbar vertebra. The values for the surface-to-volume ratios are very close (within ±4%) for sternums and their corresponding third lumbar vertebrae, reflecting the closeness of their mean trabecular path lengths. For sternums (those of 68-year-old man and 77-year-old woman) for which their matching third lumbar vertebrae were not available, data of the third lumbar vertebra of the same age group analyzed by Darley[10] have been used for comparison, and trends similar to the above results have been found.

### Structural variations

Structural variations within the sternum: To assess structural differences to demonstrate changes between the different regions of the sternum, various parts within the sternum were analyzed separately.

A sternum was cut sagittally into half, and one of the halves was cut transversally into five portions, two from the manubrium (a and b) and three from the body of the sternum (c, d and e) [Figure 3a]. Each portion of the manubrium was further cut into five portions, and each portion of the body was cut into four sagittal slices of approximately the same thickness. The results computed for the mean trabecular (t) and marrow space (l) path lengths, percentage bone volumes (Bv) and endosteal surface-to-volume ratios (S/V), together with the between-directions (σ^2^ d) and between-sections (σ^2^ S) variances for individual portions are given in Table 2.

**Table 2:** Biological variations within the sternum

*Site*	*Trabeculae*			*Marrow spaces*			*S/V*	
			
	*t ± S.E. (μm)*	*σ^2^d*	*σ^2^S*	*t±S.E.(μm)*	*σ^2^d*	*σ^2^S*	*Bv*	*cm-1*
a	181 ±8	398	670	1290 ±28	7460	10068	11.5	245
b	211 ± 10	1254	239	1801 ±85	2694	5768	10.1	199
c	188 ±9	257	752	1827 ±92	57721	70415	9.4	220
d	144 ±6	194	188	2406 ± 94	105185	23901	4.9	322
e	153 ± 10	260	1458	2168 ±75	28636	44627	5.7	302

**Figure 5:**
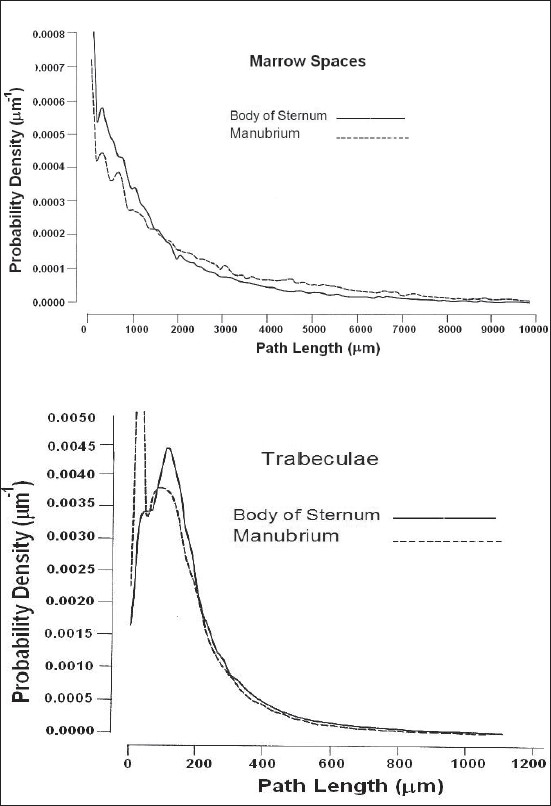
Omnidirectional path-length distributions, human adults' sternum

The general trend is for the mean trabecular path lengths to decrease as the mean marrow space path lengths increase from the jugular notch toward the xiphi-sternal junction. Consequently, the percentage bone volume decreases and the surface-to-volume ratio increases over the length of the sternum. In order to reveal the structural differences between the manubrium and the body of the sternum the structural parameters for both parts are given in Table 3, and their omnidirectional path-length distributions for marrow spaces and trabeculae are shown in Figure 5.

**Table 3:** Biological variations in manubrium and body of the sternum

*Site*	*Trabeculae*			*Marrow spaces*			*S/V*	
			
	t ± S.E. (μm)	σ^2^d	σ^2^S	ℓ ±S.E.(μm)	σ^2^d	σ^2^S	*Bv*	*cm-1*
Manubrium	193 ±7	689	505	1500 ±62	1180	74195	10.9	227
Body	170 ±6	233	799	2098 ±67	44867	70807	7.0	260
Whole Sternum	183 ±5	382	914	1825 ±61	14290	142176	8.8	241

**Figure 6:**
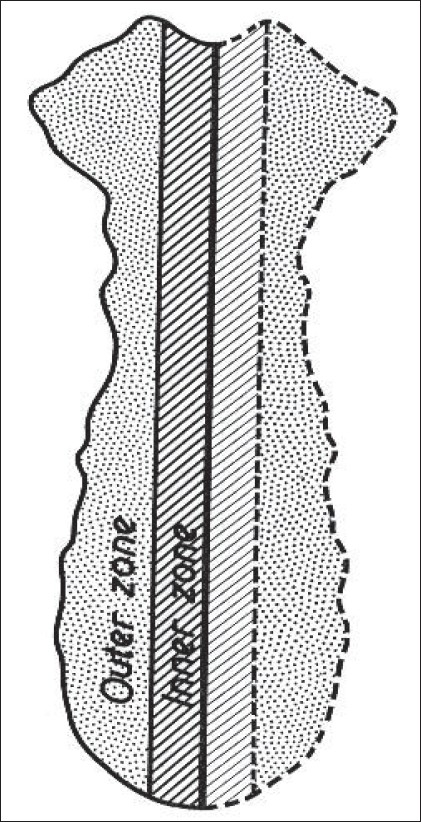
Schematic division of the sternum into inner and outer zone

The results indicate that trabecular thicknesses are larger and marrow spaces are smaller in the manubrium than the body of the sternum, consequently giving higher percentage bone volume to the manubrium (∼56%), which, apart from its larger overall width and thickness, gives it more strength than the body. To analyze the structural variations of the sternum in a direction parallel to the horizontal (×) axis, the sternum was divided into two zones, outer and inner, as demonstrated in Figure 6.

**Figure 7:**
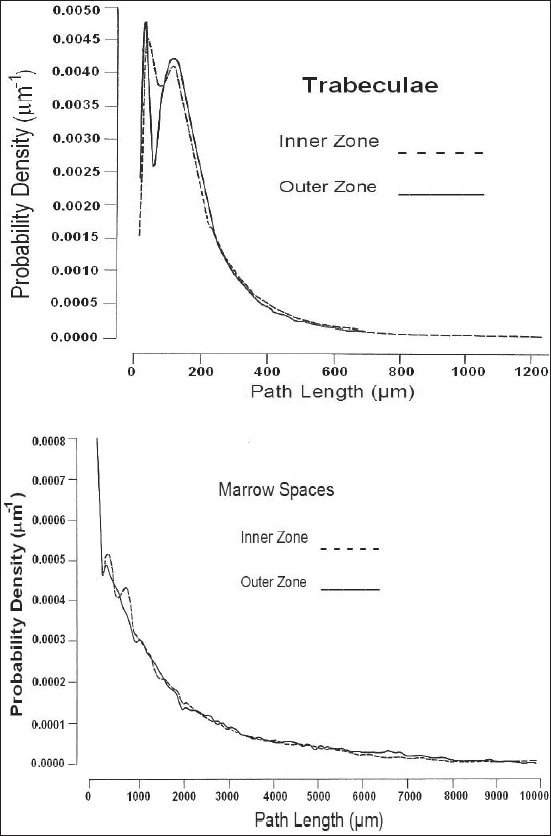
Omnidirectional path-length distributions of inner and outer zone of sternum

The omnidirectional path-length distributions of both zones for marrow spaces and trabeculae are given in Figure 7, where no significant differences are seen in their distributions.

The structural parameters for each zone are given in Table 4. In view of the errors, no appreciable difference between the inner and outer zones can be discerned.

**Table 4:** Biological variations for outer and inner zones of the sternum

*Site*	*Trabeculae*			*Marrow spaces*			*S/V*	
			
	t ± S.E. (μm)	σ^2^d	σ^2^S	ℓ ±S.E.(μm)	σ^2^d	σ^2^S	*Bv*	*cm-1*
Outer Zone	180 ± 14	390	576	1874 ± 187	15427	159964	8.4	227
Inner Zone	186 ± 17	387	1380	1780 ± 163	7502	150813	9.2	260
Combination	183 ± 13	382	914	1825 ± 128	14290	142176	8.8	241

Structural variations with age: The number of sternums analyzed is too small to allow any firm conclusion; more samples need to be analyzed if the age dependence of the structural parameters is to be determined directly.[25,26]

Intercomparisons with the lumbar vertebrae suggest that the structural parameters do not change appreciably with age.

Determination of structural anisotropy: For the purpose of characterizing trabecular bone structure or understanding the relationship between the structure and external factors acting on the bone, such as mechanical loading,[27] it is often necessary to have a means of specifying the degree and type of anisotropy present as well as other structural parameters (i.e., l, t, Bv and S/V). Anisotropy can be described in terms of the preferred orientation of features in the structure. Several means of specifying anisotropy have been suggested,[28,29,4] but they are derived from mathematical distribution functions that explicitly define the structure, and which are not found in trabecular bone.[30] The between-directions variance on the mean trabecular path lengths has been found,[7] a reasonably constant quantity in the vertebrae and from which Beddoe[13] defined an index of anisotropy. This is a useful quantity for comparison with other bones in the same individual.

The analyzed results showed that the trabecular structure exhibits less anisotropy in the sternum (0.73) than in the lumbar vertebrae (1.0).

## Results

The fact that one sagittal half of the sternum is structurally similar to the other (the coefficients of variation for mean path length and for the percentage bone volume differed by less than 5%) taken together with the symmetry of alignment about the vertical axis leads to the construction of the sectioning and scanning plans. These called for the scanning of thin, parallel, sagittal slices of half the sternum to generate the omnidirectional path-length distributions and to obtain the structural parameters for the whole sternum. The sternums were analyzed in conjunction with their corresponding third lumbar vertebrae. This enabled comparison with the results on lumbar vertebrae obtained by others.[10,13] This comparison also justified the reproducibility of the system employed and reliability of the results obtained for the sternum. In all sternum cases studied, the omnidirectional path-length distribution curves for the trabeculae take the form of an asymmetrical curve with a tail at high path-length values. These curves do not in general fit a simple distribution function. The shape and position of the curves are virtually identical for males and female of different ages, indicating that the structure arrangement of the trabeculation is similar in sternums although the absolute dimensions of trabeculae and marrow spaces may vary between sexes and for different ages. Thickness of most (∼90%) of the trabecular structures is less than 400 *μ*m, and about 65% of them fall within the range 40-200 *μ*m. A few thicker structures are present at all ages, giving the tail to each of the distribution curves. The omnidirectional path-length distribution curves for the marrow spaces are also asymmetrical, again with tail larger path lengths. Some differences were found between sexes and for different ages. Most (∼90%) of the marrow space path lengths are less than 3500 *μ*m, and about 65% of the fall within 100-1500 *μ*m. With increasing age no significant change was observed in the mean trabecular path lengths. The mean marrow space path lengths did not show an increase with age in adults, but the variations between individuals were slightly (∼2%) higher than mean trabecular path lengths. The study of whole bone, however, demonstrates that considerable structural variations exist between slices taken from different regions throughout the whole volume of the sternum.
